# Bacteriocinogenic properties of lactic acid bacteria isolated from Bulgarian feta cheese and Brazilian *prato* cheese against *Listeria monocytogenes*: Bacteriocin, an ally in the control of listeriosis

**DOI:** 10.1007/s10482-026-02335-5

**Published:** 2026-05-12

**Authors:** Kayque Ordonho Carneiro, Marcos Vinício Alves, Svetoslav Dimitrov Todorov

**Affiliations:** 1 ProBacLab, Laboratório de Microbiologia de Alimentos, Departamento de Alimentos E Nutrição Experimental, Food Research Center, Faculdade de Ciências Farmacêuticas, Universidade de São Paulo, São Paulo, SP 05508-000 Brazil; 2 CISAS - Center for Research and Development in Agrifood Systems and Sustainability, Instituto Politécnico de Viana Do Castelo, 4900-347 Viana Do Castelo, Portugal

**Keywords:** Lactic acid bacteria, Cheese, Bacteriocin

## Abstract

This study explores the bacteriocinogenic properties of *Lacticaseibacillus paracasei*, *Lactiplantibacillus plantarum*, *Pediococcus pentosaceus*, *Latilactobacillus curvatus*, and *Pediococcus acidilactici* against *Listeria monocytogenes,* strains isolated from Bulgarian feta cheese and Brazilian *prato* cheese. These bacteriocinogenic strains, when subjected to genotypic tests, showed the presence of the *ped*PA-1 gene related to pediocin synthesis. It was observed the stability of these produced bacteriocins when exposed to a wide range of temperatures, pH, and chemicals, maintaining their antimicrobial activity against different serovars of *L. monocytogenes*. The supernatant containing bacteriocin from the strains studied here inhibited the growth of different strains of *L. monocytogenes* during 9h of incubation. Regarding the safety of the studied strains, in addition to physiological and biochemical tests, only 3 of 24 genes related to vancomycin resistance and virulence were found in the strain’s DNA, with *IS*16 in *Lbs. paracasei* ST0110KOC and the *ace* gene in *Lpb. plantarum*.

**Supplementary Information:**

The online version contains supplementary material available at 10.1007/s10482-026-02335-5.

## Introduction

Lactic acid bacteria (LAB) are group of Gram-positive bacteria, cocci or bacilli, catalase-negative and non-spore-forming (Freire et al. 2021), plays an essential roles in fermented foods and are widely distributed in soil, water, plants, the gastrointestinal tract (GIT) of humans and other animals and in food product manufacturing environments, being divided among the genera: *Lactobacillus*, *Streptococcus*, *Leuconostoc*, *Enterococcus*, *Pediococcus* and others (Lima et al. 2024). Building scientific data and enormous variety of LAB and specially these belong to the Genera *Lactobacillus* and *Leuconostoc* justified the proposed by Zheng et al. (2020) reclassification of the mentioned genera into 25 new genera, regrouping the microorganisms according to their shared ecological and metabolic properties. Further contributing to a better representation and organization of these 25 new genera and the existing ones, Todorov et al. (2023) suggested the introduction of new abbreviations for the genera *Lactobacillus* and *Leuconostoc*. According to the suggestions of Zheng et al. (2020), all representatives from former genus *Lactobacillus* will be named according in this manuscript.

LAB are widely used in the food industry as starter cultures due to their fermentative capacity, contributing to the formulation of new products (Freire et al. 2021), where also has technological and biopreservation properties, associated with range of beneficial metabolites. These properties may contribute directly to the organoleptic characteristics and safety of food (Carneiro et al. 2024) and are directly associated with the metabolic capacity of LAB, which includes the production of lactic acid and other organic acids, diacetyl, enzymes, bacteriocins, bioactive peptides, exopolysaccharides, antioxidants, short-chain fatty acids (SCFAs), γ-aminobutyric acid (GABA), and other functionally active metabolites (Carneiro et al. 2024; Furtado et al. 2014; Gómez et al. 2016; Hill et al. 2014; Holzapfel et al. 2018; Shi et al. 2019).

It should be added that several strains belong to LAB species were suggested and evaluated as probiotic bacteria, that is, live microorganisms that, when administered in adequate amounts, confer health benefits to the host (ANVISA, 2021; FAO/WHO 2006). These LABs, when classified as probiotics, exert beneficial effects on the GIT, acting in the modulation of the immune response, strengthening the intestinal barrier and epithelial cells, as well as competing for nutrients and adhesion sites with pathogenic microorganisms. These functions contribute significantly to the balance of the intestinal microbial ecosystem, being associated with a reduction in the incidence of inflammatory, metabolic, and infectious diseases. However, the beneficial effects are specific to each strain and depend on both the administered dose and the viability of the microorganism at the time of consumption (Saad 2006).

According to Chikindas et al. (2018), all microorganisms can produce antimicrobial proteins, as they are important for conquest of the ecological niches and even are involved in intracellular communications. Antimicrobial proteins can be divided into two groups: non-ribosomal antimicrobial proteins and ribosomal antimicrobial proteins, with bacteriocins being an example of the latter group (Chikindas et al. 2018). Bacteriocins are cationic antimicrobial polypeptides produced ribosomally by bacteria. These compounds predominantly exhibit extracellular activity, with a mechanism of action largely directed at the cell membrane of target microorganisms. Due to their effective antimicrobial activity, bacteriocins have been extensively investigated and considered promising biocontrol tools, especially in the context of food safety and public health (Choi et al. 2023; Carneiro et al. 2024).

Bacteriocins are classified based on their bacterial source (Gram-positive or Gram-negative), biochemical properties, mechanism of action, chemical structure, molecular size, and thermal stability. According to these characteristics, they are grouped into classes I, II, III, and IV. Class I bacteriocins are extremely small and have a low molecular weight (< 5 kDa), formed by lanthionine and methyl-lanthionine, and are also called lantibitics. These bacteriocins exhibit thermostability and undergo post-translational modifications in the bacterial cytoplasm, also known as RiPPs (ribosomically synthesized and post-translationally modified peptides).

Class II bacteriocins are like lantibitics in size (< 10 kDa), but structurally distinct. They are composed of common amino acids, not carrying lanthionine and methyllanthionine in their structure. This class of bacteriocin is subdivided into 5 groups, differing in their structure, composition, and mode of action. Furthermore, it should be noted that class II bacteriocins are classified as unmodified or minimally modified during their maturation period. These discrete alterations may include the formation of disulfide bridges, circularization of the molecule, or the addition of N-formylmethionine, making it biologically active. Thus, bacteriocins of this class are known as non-lantibitics. Unlike class I bacteriocins, they do not undergo post-translational modifications and are not formed by the amino acids lanthionine and methyllanthionine. For this reason, they are known as non-lantibitics (Cotter et al. 2013; Alvarez-Sieiro et al. 2016). These two classes are widely studied and described in the context of bacteriocins produced by LAB. Moreover, due to their small molecular size, bacteriocins belonging to classes I and II exhibit high physicochemical stability, being less susceptible to denaturation. These molecules maintain their biological activity even under extreme temperature conditions, wide pH variations, and in the presence of chemical agents (Iseppi et al. 2019).

*Listeria monocytogenes* is a Gram-positive, facultative anaerobic pathogen with a capacity for growth over a very wide temperature range. This pathogen can survive temperatures as low as − 0.4 °C, remaining active even in conditions that inhibit other microorganisms. In cold environments, *L. monocytogenes* exhibits adaptive mechanisms that ensure its survival. Among these, the most notable is the increased proportion of unsaturated fatty acids in the cell membrane, which preserves its fluidity by altering its lipid composition (Domingues et al. 2025). Furthermore, this pathogen remains motile at temperatures of 22–28 °C, with an optimal growth temperature of 37 °C (Osek et al. 2022). *L. monocytogenes* is of great concern in the food industry because it can multiply over a very wide temperature range from − 0.4 °C to 45 °C, surviving in environments with low water activity (aW < 0.90), pH between 4.6 and 9.5, and sodium chloride concentrations above 20% (Bucur et al. 2018). Thus, *L. monocytogenes* exhibits a capacity for survival under very wide extreme conditions, being able to multiply under conditions commonly used for food preservation (Gray et al. 2006). Furthermore, *L. monocytogenes* can establish themselves on biotic and abiotic surfaces due to its ability to form biofilms (Osek et al. 2022). The biofilm consists of a barrier composed of a cluster of microorganisms, water, extracellular polymers, and proteins. This barrier hinders the action of agents commonly used in environmental sanitation and food preservation, thus allowing the permanence of resident bacteria, conferring greater resistance to them (Lima et al. 2024).

Due to its broad resistance and high virulence, *L. monocytogenes* is an important pathogen associated with foodborne illnesses (FBIs). Listeriosis, a disease caused by this pathogen, poses a great risk to immunocompromised individuals, pregnant women, the elderly, newborns, and recent transplant recipients, presenting a significant mortality rate of 20–30% worldwide (Buchanan et al. 2017). However, certain bacteriocins produced by LAB exhibit antimicrobial action against strains of *L. monocytogenes*, a property of extreme importance for the control of this pathogen (Rwubuzizi et al. 2023; Lima et al. 2024). Rwubuzizi et al. (2023) were able to verify bacteriocinogenic activity of species of *Lpb. plantarum*, *Lct. curvatus*, *Lbs. paracasei*, *P. pentosaceus*, *Leuconostoc mesenteroides* and *E. faecium* against *L. monocytogenes*. Lima et al. (2024) were also able to observe bacteriocinogenic activity of *E. faecium* and *Lc. garvieae* against different serovars of *L. monocytogenes*.

The present research aimed to evaluate the safety of each LAB strain isolated from Bulgarian feta cheese and Brazilian *prato* cheese and its bacteriocinogenic capacity against different serovars of *L. monocytogenes*, as well as the stability of the bacteriocin produced under the different physicochemical conditions explored.

## Materials and methods

### Isolation and screening of bacteria potentially producing bacteriocins

Previously isolated and preliminary identified as bacteriocin producing LAB from Bulgarian Feta Cheese and some additional isolated potential bacteriocinogenic strains from Brazilian *prato* cheese following same experimental procedures (Carneiro et al. 2026) were further investigated as their potential for control of *L. monocytogenes*. In summary, the isolates obtained after implementing 3 levels approach for isolation of potential producers of bacteriocins (Rwubuzizi et al. 2023) were cultured in MRS broth (Difco) for 24h at 37 °C, subjected to morphological analysis, Gram staining, and catalase reaction as described by de Vos et al. (2009), and further confirmed as bacteriocin producer and followed by storage in cryogenic tubes containing 20% glycerol (v/v) and frozen at -20 °C for further analysis.

### Supernatant containing bacteriocin and inhibition spectrum

The catalase-negative and Gram-positive isolates were chosen after screening for bacteriocin-producing LAB. The selected isolates were subsequently tested for bacteriocin production using a modified version of the method described by Valledor et al. (2022) and dos Santos et al. (2020). The isolates were activated in MRS broth (Difco) for 24h at 37 °C, then centrifuged (10,000 × g, 5 min., 4 °C), pH adjusted between 5.5 and 6.5 using sterile 3 M NaOH and filtered through 0.22 µm filter syringes (Kasvi^®^, São José dos Pinhais, SP, Brazil), being transferred to a sterile 1.5 mL Eppendorf tube. These aliquots were treated for 10 min. at 80 °C to reduce the potential effects of proteases or H_2_O_2_. Then, 10 μL of each treated aliquot was distributed using the spot-on-the-lawn technique (dos Santos et al. 2020) on BHI plates supplemented with 1% agar (w/v) individually containing *Salmonella enteritidis* ATCC 13076., *Klebsiella aerogenes* ATCC 13048, *Staphylococcus aureus* ATCC 29213, *Bacillus cereus* ATCC 11778, *Escherichia coli* ATCC 8739, *Clostridium perfringens* ATCC 13124, *L*. *monocytogenes* ATCC 7644, *L*. *monocytogenes* 408 serovar 1/2c, *L*. *monocytogenes* 103 serovar 1/2a, *L*. *monocytogenes* 409 serovar 1/2a, *L*. *monocytogenes* 302 serovar 4b, *L*. *monocytogenes* 620 serovar 4b, *L*. *monocytogenes* 211 serovar 4b, *L*. *monocytogenes* 106 serovar 1/2a, *L*. *monocytogenes* 506 serovar 1/2a, *L*. *monocytogenes* 422 serovar 1/2c, *L. monocytogenes* 603 serovar 1/2b, *L*. *monocytogenes* 101 serovar 4b, *L*. *monocytogenes* 712 serovar 1/2c e *L*. *monocytogenes* 724 serovar 4b (isolated from the collection of the Food Microbiology Laboratory of Faculty of Pharmaceutical Sciences, University of Sao Paulo, São Paulo, SP, Brazil) at an approximate concentration of 10^5–6^ CFU/mL. The plates were incubated for 24h at 37 °C and inhibition zones were observed. Inhibition zones of at least 3 mm were considered indicative of bacteriocin production, and further activities were subsequently performed.

### Differentiation and identification of the isolates

The isolated LAB with bacteriocinogenic characteristics were differentiated as reported previously and identified based on morphological, physiological and biochemical recommendations according to de Vos et al. (2009) and 16S rRNA partial sequencing. In summary selected isolated were grown in 9 mL of MRS broth (Difco) for 24h at 37 °C. Subsequently, the DNA of each isolate was extracted using a commercial Quick-DNA Fungal/Bacterial Miniprep Kit (Zymo Research, Irvine, CA, USA), according to the manufacturer's recommendations. The concentration and quality of the obtained DNA were evaluated in a nano-spectrophotometer (NanoDrop-Thermo Fisher Scientific, Waltham, MA, USA) and adjusted to approximately 50 ng/µL, and the differentiation and identification reactions were performed afterwards. The differentiation process of the isolates consisted of performing *rep*PCR using the primer (5′GTG)_5_–3′) and conditions described according to de Castilho et al. (2019a). Furter, identification was performed used to target the conserved ribosomal regions (16s), with primers BSF8/20- F AGAGTTTGATCCTGGCTCAG and BSR 1541/20-R AGGAGGTGATCCAGCCGCA and conditions recommended by Héquet et al. (2007) (Supplementary Table 1). Subsequently, the amplicons were sequenced by a third-party service at the Institute of Biomedical Sciences, University of São Paulo, Human Genome and Stem Cell Research Center using the Sanger DNA sequencing technique (BigDye Terminator v3.1 Cycle Sequencing Kit) and identified by Sequencing Analysis 7.0 (Base Caller KB™). The received sequences were then analyzed using the Basic Local Alignment Search Tool (BLAST, National Center for Biotechnology Information, Bethesda, MD, USA).

### Safety assessment of LABs

#### Hemolytic activity

To perform the hemolytic activity assay, the recommendations of Fugaban et al. (2021a) were followed. The strains were previously cultured in 9 mL of MRS broth (Difco) for 24h at 37 °C, subcultured onto plates containing Sheep Blood Agar medium (Laborclin, SP, Brazil), and incubated for 24h at 37 °C. The plates were observed for halo formation, and the absence of halos around the colonies was considered indicative of strains without hemolytic activity (γ-hemolytic). Colonies with halo formation were considered α-hemolytic (green halos) or β-hemolytic (translucent halos), evaluated as positive for hemolysis and excluded from the study. *L. monocytogenes* ATCC 7644 was used as a positive control for α-hemolytic activity.

### Production of biogenic amines

The evaluation of biogenic amine production was performed using the methodology described by Bover-Cid and Holzapfel (1999). Initially, the selected strains were cultured in MRS broth supplemented with precursors for biogenic amine production in proportions of 1:100 (tyrosine, ornithine, lysine, and histidine, all from Sigma-Aldrich) and 0.005% pyridoxal-5-phosphate (w/v) (Sigma-Aldrich) for 24h at 37 °C for 5 subsequent times. Plates with MRS (Difco) were supplemented with 2% agar (w/v) and specific precursors for biogenic amine production were prepared. Subsequently, all plates were incubated for 7 days at 37 °C and then observed. A change in color from yellow to violet represented decarboxylation of the biogenic amine precursor supplements, and strains exhibiting this behavior were classified as positive and excluded. *B. cereus* ATCC 11778, *Lpb. plantarum* ST8Sh, and *L. monocytogenes* ATCC 7644 served as controls.

### Resistance to antibiotics

The previously incubated isolates were subjected to antibiogram testing, following the antibiotic selection recommendations as described by EFSA (2018) with minimal inclusions and obtained results interpreted according to CLSI. The test itself was performed according to the methodology described by Rwubuzizi et al. (2023). Sterile plates received 1 mL of sterile saline solution (0.85% NaCl, w/v), 40 μl of the isolate at an initial concentration of 10^6–7^ CFU/mL, and 40 mL of MRS agar (Difco). Subsequently, antibiotic diffusion discs were placed on the agar and incubated for 24h at 37 °C. The antibiotics tested were vancomycin (30 µg/disc), streptomycin (10 µg/disc), gentamicin (10 µg/disc), ofloxacin (5 µg/disc), ampicillin (20 µg/disc), chloramphenicol (30 µg/disc), erythromycin (15 µg/disc), tylosin (60 µg/disc), amoxicillin (10 µg/disc), tetracycline (30 µg/disc) and kanamycin (30 µg/disc), all from Cefar (Cefar Diagnóstica Ltda, São Paulo, Brazil). Nalidixic acid (30 µg/disc) (Cefar) was used as a negative control, as it only has activity against Gram-negative species.

### Evaluation of the genes present in the DNAs of the selected strains

#### Beneficial genes

The analysis of the presence of beneficial mucosal adhesion genes (*map*, *mub* and *eftu*), aggregation substance gene (*prg*), adhesion proteins (*EF*2662, *EF*1249, *EF*2380) and folate synthesis genes (*pab*B, *pab*C, *fol*KQ and *fol*PE) was performed according to de Castilho et al. (2019b) and Kim et al. (2022) (Supplementary Table 1). Each PCR reaction was performed in a Veriti 96 thermal cycler, following the recommendations described by each author. The amplicons were separated on a 1.5% (w/v) agarose gel by electrophoresis (100 V, 45 min.). Subsequently, the gels were stained with SYBR^®^ Safe DNA Gel Stain (Thermo Scientific) and visualized in a Molecular Imager^®^ GelDoc™ XR (Bio-Rad). The results were evaluated according to the size of each target amplicon.

### Bacteriocin genes

The presence of the genes *ent*A, *ent*B, *ent*L50B, *ent*P, *ped*PA-1, *plc*A, *lgn*A, *lgal*, *gak*, *gak(R1)*, lcn972 and *lcn-gq*, related to bacteriocin production, was investigated in the DNA of each LAB strain. Each PCR reaction was performed in a Veriti 96 thermal cycler, following the recommendations described by Fugaban et al. (2021b), Todorov et al. (2010), Maldonado Barragán et al. (2013), Telke et al. (2019), and Mirkovic et al. (2015) (Supplementary Table 1). The amplicons were separated on agarose gel (1–2%, w/v) by electrophoresis (100 V, 45 min.). Subsequently, the gels were stained with SYBR^®^ Safe DNA Gel Stain (Thermo Scientific) and visualized in a Molecular Imager^®^ GelDoc™ XR (Bio-Rad). The results were evaluated according to the size of each target amplicon.

### Virulence genes

The DNA of each strain was analyzed for the presence of virulence-related genes, namely *van*A, *van*B, *van*C, *van*D, *van*E, *van*G, *IS*16, *ace*, *efa*, *esp*, *asa*, *hyl*, *hdc*, *tdc*, *odc*, *cyl*A and *gel*. PCR reactions were performed in a Veriti 96 thermal cycler, as recommended by each author related to the gene of interest according to Fugaban et al. (2021a), Werner et al. (2011), Martín Platero et al. (2009), Vankerckhoven et al. (2004) de Las Rivas et al. (2005) (Supplementary Table 1). The amplicons were separated on agarose gel (1–2%, w/v) by electrophoresis (100 V, 45 min), the gels were supplemented with SYBR^®^ Safe DNA Gel Stain (Thermo Scientific) and visualized on a Molecular Imager^®^ GelDoc™ XR (Bio-Rad). The results were evaluated considering the size of the target amplicons.

### Mucin degradation

The strains were pre-activated in 9 mL of MRS broth (Difco) for 24h at 37 °C, followed by the mucin degradation test according to Rwubuzizi et al. (2023). The MRS culture medium was prepared manually according to the commercial composition, supplemented with 1.5% agar (w/v), 0.3% MGP (w/v) (porcine gastric mucin, type III, Sigma-Aldrich), both without and with 1% glucose. Both prepared media were autoclaved for 15 min at 121 °C and distributed into sterile plates. Ten microliters of each strain were inoculated onto the surface and incubated for 72h at 37 °C. Subsequently, the colonies were stained for 30 min with 0.1% amino black (Sigma Aldrich) (w/v) in 3.5 M acetic acid (Synth). Finally, the plates were washed with 1.2 M acetic acid and the lytic zones around the colonies were evaluated for degradation capacity. *B. cereus* ATCC 11778 was used as positive control.

### Characterization of bacteriocins

#### Validation of the protein nature of bacteriocins

The supernatants of potential bacteriocin-producing strains were selected and prepared as previously described following the methodology described by Valledor et al. (2022). The supernatants were individually treated with 0.1 mg/mL of Proteinase K (final concentration), incubated for 1h at 37 °C, followed by heat treatment for 3–5 min at 98–100 °C to stop the enzymatic reaction. Then, the residual antimicrobial activity, measured in AU/mL (Arbitrary Units), was evaluated against *L. monocytogenes* 211 serovar 4b (L211), *L. monocytogenes* 422 serovar 1/2c (L422) and *L. monocytogenes* 603 serovar 1/2b (L603) under previously described conditions. The supernatant not treated with Proteinase K served as a positive control.

### Analysis of bacteriocin stability

The stability assessment of the bacteriocins followed the methodology described by dos Santos et al. (2020), which aimed to expose the bacteriocins present in the previously treated supernatants to different temperature, pH, and chemical conditions, conditions present in industries and laboratories. For temperature variation, the supernatants were incubated at 8 °C, 30 °C, 37 °C, 60 °C, and 98 °C for 1h and 120 °C for 15 min. For the influence of selected chemicals, the supernatants were exposed to 10 mg/mL of NaCl, SDS, Tween 80, Tween 20, and skim milk for 1 h at 37 °C. Finally, for pH variation, the pH of the supernatants was adjusted with sterile 1 M NaOH or 1 M HCl to levels of 2.0, 4.0, 6.0, 8.0, or 10.0. The samples were incubated for 1 h at 37 °C, and the pH was readjusted to 5.0–7.0 with 1 M NaOH or 1 M HCl, as needed. After incubation, all experimental variations were tested for residual bacteriocin activity against *L. monocytogenes* 211, 422, and 603, following the same incubation method described previously, and AU/mL were determined following the mathematical formula described by Valledor et al. (2022): dilution type (**D** = 2x), first dilution that did not show an inhibition zone (n), and inoculated volume (**P** = 10 μL), represented below. The untreated supernatant served as a positive control for inhibition.

$$\frac{AU}{{mL}} = \frac{{D^{n} x 1000}}{P}$$

### Growth kinetics, acidification, and bacteriocinogenic activity of LAB

Based on the *rep*PCR, bacteriocinogenic strains were selected, and subjected to growth kinetics, acidification, and bacteriocin production according to Valledor et al. (2022). The strains were incubated at 5% (v/v) for 24h at 37 °C in MRS broth (Difco) from strains previously activated for 18h. The growth of the strains was evaluated every 1h based on the change in optical density (OD), measured at 600 nm in an Ultrospec 2000 (Pharmacia Biotech, England), simultaneously with the recording of pH change in a pH meter (Láctea Aparelhos Científicos e Eletrônicos LTDA, São Paulo, Brazil). Furthermore, aliquots were collected every 3h to assess bacteriocin concentration. The samples were serially diluted 2 × in 100 mM potassium phosphate buffer (pH 6.5) and 10 μL volumes were inoculated onto plates containing *L. monocytogenes* 211, 422, and 603 (final concentration 10^5–6^ CFU/mL), as described previously. The bacteriocinogenic activity capacity was expressed in AU/mL, following the mathematical formula as described in the previous section.

### Kinetics of *L. monocytogenes *cell death induced by treated supernatants containing bacteriocins

The supernatant obtained from 24h cultures of studied bacteriocinogenic strains grown at 37 ºC was treated as previously described and added to L211, L422, and L603 cultures in the early exponential growth phase. Initially, the aforementioned *L. monocytogenes* cultures were inoculated into 200 mL of BHI broth (Oxoid) with a 1% (v/v) inoculum and incubated at 37 °C. The treated supernatants from the selected bacteriocinogenic strains were filtered through a 0.22 μm filter (Kasvi®) and added to the *L. monocytogenes* 211, 422, and 603 cultures after 3h of incubation. Changes in OD at 600 nm were analyzed every 1h using an Ultrospec 2000 spectrophotometer (Pharmacia Biotech) along for 12h. *L. monocytogenes* 211, 422, and 603 cultures without supernatant addition were used as a growth control (Favaro et al. 2014).

## Results and discussion

### Isolation and screening of potentially bacteriocinogenic bacteria

In a previous study (Carneiro et al. 2026) based on preliminary application of 3 levels approach for isolation of bacteriocinogenic LAB, a set of 11 isolated were preselected for further investigation. In addition, 2 strains from Brazilian *prato* cheese were isolated and considered for further investigation. Based on performed repPCR differentiation and following 16S rRNA partial gene sequencing the 13 isolates were identified as *Pediococcus pentosaceus*, *Latilactobacillus curvatus*, *Pediococcus acidilactici*, *Lactiplantibacillus plantarum* and *Lacticaseibacillus paracasei* (Carneiro et al. 2026).

**Fig. 1:**
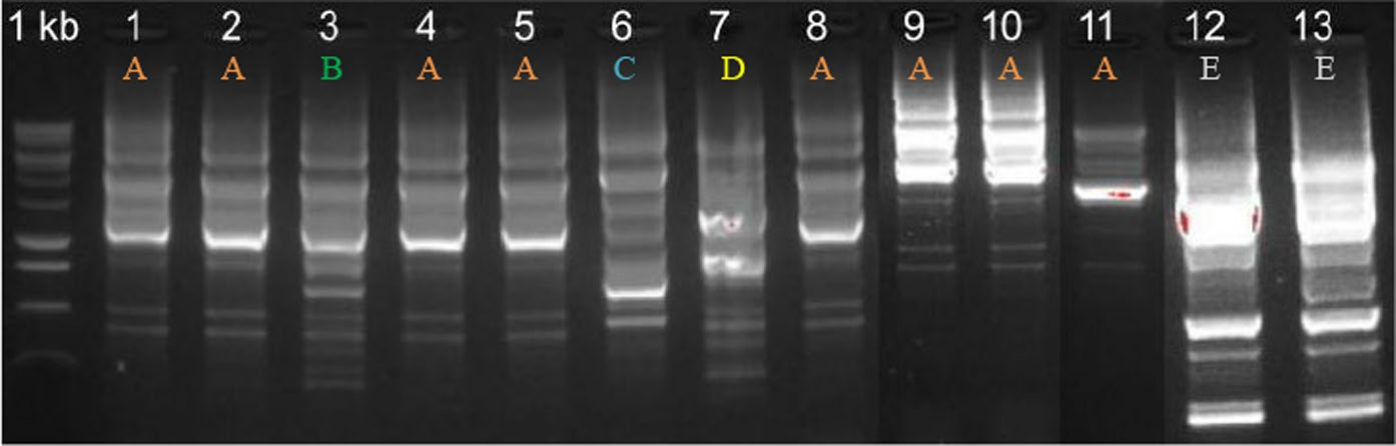
Agarose gel electrophoresis of isolates 1 to 13, differentiated by *rep*PCR. Groups: **A**. *Pediococcus pentosaceus*; **B**. *Latilactobacillus curvatus*; **C**. *Pediococcus acidilactici*; **D**. *Lactiplantibacillus plantarum*; **E**. *Lacticaseibacillus paracasei*. O’GeneRuler™ 1 kb DNA Ladder (Fermentas) were utilized

*Lbs.* paracasei (ST0110KOC and ST0158KOC) was isolated from Brazilian *Pprato* cheese and was the only species detected exclusively in this sample among all those analysed. The genera of the listed bacteria are commonly considered safe (GRAS) and their presence ends up being satisfactory in food production (commonly classified as having a qualified presumption of safety-QPS), due, in their great majority, to their ability to produce antimicrobial metabolites, which are applied as biopreservatives (Margalho et al. 2020). In samples 2, 3 and 4 of Bulgarian feta cheese, a diversity in their microbiota was observed, carrying the following bacteria of distinct genera and species: *Lpb*. *plantarum* (ST0414KOC), *P. pentosaceus* (ST0401KOC, ST0402KOC, ST0406KOC, ST0407KOC, ST0408KOC, ST0410KOC, ST0415KOC, ST0420KOC), *Latilactobacillus curvatus* (ST0403KOC), and *Pediococcus acidilactici* (ST0412KOC) (Fig. 1, Supplementary Table 2). Specifically, sample 4 showed a predominance of the species *P. pentosaceus*, a species commonly used as a starter culture in food production due to its metabolic ability, generally accompanied by its bacteriocinogenic activity (Todorov et al. 2022). For example, it is used in cheese production (Cavicchioli et al. 2019).The focus of the investigation was narrowed down to the strains of *Pediococcus pentosaceus* ST0401KOC, *Latilactobacillus curvatus* ST0403KOC, *Pediococcus acidilactici* ST0412KOC, *Lactiplantibacillus plantarum* ST0414KOC and *Lacticaseibacillus paracasei* ST0110KOC to be discussed.

LAB are a community present in various types of food: fermented vegetables (Rwubuzizi et al. 2023), sausages (Carneiro et al. 2024), dairy drinks and yogurts (Sousa et al., 2022), cheeses (Cavicchioli et al. 2019; Neto et al. 2005), and countless other foods. In cheeses, it is possible to identify a variety of genera and species of this non-taxonomic group: *Pediococcus* spp. (Cavicchioli et al. 2019), *Lactobacillus* spp. (Neto et al. 2005), *Enterococcus* spp. (Favaro et al. 2014), *Streptococcus* spp. (Franciosi et al. 2015).

Next, the selected individual isolated were subjected to Gram and catalase tests for selectivity of LAB, follow by a preliminary safety test for vancomycin resistance and β-hemolytic or α-hemolytic activity. The selected for further study strains were evaluated for their antimicrobial activity versus *S. enteritidis* ATCC 13076, *K. aerogenes* ATCC 13048, *Staph. aureus* ATCC 29213, *B. cereus* ATCC 11778, *E. coli* ATCC 8739, *C. perfringens* ATCC 13124, *L*. *monocytogenes* ATCC 7644, *L. monocytogenes* 408 serovar 1/2c, *L*. *monocytogenes* 103 serovar 1/2a, *L*. *monocytogenes* 409 serovar 1/2a, *L*. *monocytogenes* 302 serovar 4b, *L*. *monocytogenes* 620 serovar 4b, *L*. *monocytogenes* 211 serovar 4b, *L*. *monocytogenes* 106 serovar 1/2a, *L*. *monocytogenes* 506 serovar 1/2a, *L*. *monocytogenes* 422 serovar 1/2c, *L*. *monocytogenes* 603 serovar 1/2b, *L*. *monocytogenes* 101 serovar 4b, *L. monocytogenes* 712 serovar 1/2c and *L*. *monocytogenes* 724 serovar 4b. Those strains that showed a broad spectrum of inhibition against one or more pathogens were selected for further testing.

Sample 1 of Brazilian *prato* cheese had only two isolates with antimicrobial action (ST0110KOC and ST0158KOC), as well as being the only ones with bacteriocinogenic activity against *K. aerogenes* ATCC 13048, which, in turn, after complete genome sequencing, proved to be more closely related to *Klebsiella pneumoniae*, and was therefore reclassified (Tindall et al. 2017). In addition, it is known that these species are responsible for numerous intra-abdominal, respiratory, urinary, blood tissue and wound infections, as well as intrinsic resistance to β-lactams, antibiotics of wide clinical use, resulting in therapeutic limitations (Choi et al. 2008; Jeon et al. 2021). Due to their characteristics and limited tools for their control, the antimicrobial capacity of isolates ST0110KOC and ST0158KOC has potential in controlling *K. pneumoniae* but requires further investigation. In addition, isolate ST0158KOC was the only one with antimicrobial activity against *S. enteritidis* ATCC 13076, recognized for its ability to establish itself in biotic and abiotic environments (Chylkova et al. 2017) and of great sanitary concern, in which it was responsible in 2013 for 39.5% of the 82.694 cases of salmonellosis, generating more than 7.800 hospitalizations (EFSA and ECDC, 2015). Added to its clinical attention, *Salmonella* species are widely present in cases of cross-contamination, aided by their ability to form biofilms (Kim; Kim, 2022; Yang et al. 2016). The antimicrobial capacity of ST0158KOC against *S. enteritidis* ATCC 13076 should be further investigated for possible use as a technological biocontrol agent.

Among the isolates obtained, only those from samples 1 and 4 were able to present well-defined inhibition zones against different types of *L. monocytogenes* serovars, namely: isolates from *prato* cheese ST0110KOC and ST0158KOC corresponding to sample 1, and 11 isolates from Bulgarian feta-type cheese ST0401KOC, ST0402KOC, ST0403KOC, ST0406KOC, ST0407KOC, ST0408KOC, ST0410KOC, ST0412KOC, ST0414KOC, ST0415KOC and ST0420KOC. These isolates from sample 5 showed high antimicrobial activity against numerous serovars of *L. monocytogenes* as shown in Table 1.

**Table 1:** Spectrum inhibition of bacteriocinogenic isolates against different pathogen

**Isolates**	*L. monocytogenes* 302 serovar 4b	*L. monocytogenes* 620 serovar 4b	*L. monocytogenes* 211 serovar 4b	*L. monocytogenes* 106 serovar 1/2a	*L. monocytogenes* 506 serovar 1/2a	*L.* *monocytogenes* 422 serovar 1/2c	*L.* 603 serovar 1/2b	*L.* monocytogenes 101 serovar 4b	*L.* *monocytogenes* 712 serovar 1/2c	*L. monocytogenes* 724 serovar 4b
ST0401KOC	** + **	** + **	** + **	** + **	** + **	** + **	** + **	** + **	** + **	** + **
ST0402KOC	** + **	** + **	** + **	** + **	** + **	** + **	** + **	** + **	** + **	** + **
ST0403KOC	** + **	** + **	** + **	−	−	** + **	** + **	** + **	** + **	** + **
ST0406KOC	** + **	−	** + **	−	−	−	−	−	−	−
ST0407KOC	** + **	** + **	** + **	** + **	** + **	** + **	** + **	** + **	** + **	** + **
ST0408KOC	−	−	** + **	−	−	** + **	** + **	−	−	−
ST0410KOC	** + **	** + **	** + **	** + **	** + **	** + **	** + **	** + **	** + **	** + **
ST0412KOC	** + **	** + **	** + **	** + **	** + **	** + **	** + **	** + **	** + **	** + **
ST0414KOC	−	−	−	−	−	−	−	−	−	−
ST0415KOC	−	−	−	** + **	−	−	−	−	−	−
ST0420KOC	** + **	** + **	** + **	−	** + **	** + **	** + **	** + **	** + **	** + **
ST0110KOC	−	−	** + **	−	−	** + **	** + **	−	−	−
ST0158KOC	−	−	−	−	−	−	−	−	−	−

**Isolates**	*L. monocytogenes* ATCC 7644	*L. monocytogenes* 408 serovar 1/2c	*L. monocytogenes* 103 serovar 1/2a	*L. monocytogenes* 409 serovar 1/2a	*C. perfringens* ATCC 13124	*S. enteritidis* ATCC 13076	*K. aerogenes* ATCC 13048	*S. aureus* ATCC 29213	*B. cereus* ATCC 11778	*E. coli* ATCC 8739
ST0401KOC	−	** + **	** + **	** + **	−	−	−	−	−	−
ST0402KOC	−	** + **	** + **	** + **	−	−	−	−	−	−
ST0403KOC	−	−	−	−	−	−	−	−	−	−
ST0406KOC	−	** + **	−	−	−	−	−	−	−	−
ST0407KOC	−	** + **	** + **	** + **	−	−	−	−	−	−
ST0408KOC	−	−	−	−	−	−	−	−	−	−
ST0410KOC	−	** + **	** + **	** + **	−	−	−	−	−	−
ST0412KOC	** + **	** + **	** + **	** + **	−	−	−	−	−	−
ST0414KOC	−	−	−	−	−	−	−	−	−	−
ST0415KOC	−	−	−	−	−	−	−	−	−	−
ST0420KOC	−	** + **	** + **	** + **	−	−	−	−	−	−
ST0110KOC	** + **	** + **	−	−	−	−	** + **	−	−	−
ST0158KOC	** + **	−	−	−	−	** + **	** + **	−	−	−

Thus, the isolates obtained in this study provide alternatives as possible biocontrol, since *L. monocytogenes* is considered a high risk to public health (Acciari et al. 2016) due to its widespread presence in biotic and abiotic environments (Lima 2024). Among the *L. monocytogenes* used, strains 211, 422, and 603 were affected by a greater number of isolates, and these were selected for further analysis.

Taking into consideration *rep*PCR (Carneiro et al. 2026) and preliminary results of the spectrum of activity of studied strains, 5 strains (*P. pentosaceus* ST0401KOC, *Lct. curvatus* ST0403KOC, *P. acidilactici* ST0412KOC, *Lpb. plantarum* ST0414KOC and *Lbs. paracasei* ST0110KOC) were selected for future investigation.

### LAB security

The discovery of new strains should be valued beyond their beneficial properties. It is of utmost importance that the safety investigation process be analyzed individually for each strain studied, since the use of these strains is intended to be explored in human and veterinary applications (for example, as probiotics) (Roe et al. 2022). Although some species are traditionally considered safe, have a long history of use without relevant incidents, or have GRAS status, these arguments do not guarantee that all their strains are safe. This insecurity and increased attention can be explained by the numerous interactions with the environment, the development of antimicrobial resistance (antibiotics and biocides), genetic alterations, and mutations (Churin et al. 2026). These negative abilities can be acquired even in species commonly considered safe, due to excessive exposure to antimicrobials, environmental changes, and also interactions between microbial communities, with horizontal transfer of genetic fragments occurring between genera and species (Endale et al. 2023).

Thus, the 5 strains selected were analyzed for their safety through different phenotypic analyses. None of the strains showed hemolytic activity and were classified as γ-hemolytic. Strains, to be considered safe, should not produce hemolysin, an exotoxin that degrades the red blood cell membrane, causing cell rupture and serious health problems (Rwubuzizi et al. 2023). These negative properties are found in pathogenic bacteria, which are classified as α and β-hemolytic. Therefore, the hemolytic activity test, besides being simple and exclusionary, requires strains to be considered safe to exhibit γ-hemolytic activity, that is, without the capacity for degradation (Jeong and Lee 2015). Similar results for γ-hemolytic LAB were obtained by Colombo et al. (2020) with strains of *Lacticaseibacillus casei*, *Lactobacillus acidophilus*, *Lpb. plantarum*, *P. pentosaceus*, *P. acidilactici* and others. Borges et al. (2013) also found an absence of hemolytic activity in a strain of *P. pentosaceus*. These results reinforce the prevalence of BAL safety based on its γ-hemolytic classification.

After performing an antibiogram, all strains selected showed sensitivity to more than 6 antibiotics, with ampicillin, chloramphenicol, erythromycin, tylosin, amoxicillin, and tetracycline being the most prevalent. These results are promising, given that the indiscriminate use of antimicrobials, as well as the ability of different species to interact, presents an opportunity for microorganisms previously considered safe to express virulent characteristics of antibiotic resistance. This is possible due to the horizontal transfer of genetic information between bacteria, extending to the transfer of other genetic fragments related to toxin production. Therefore, it is worth noting that, to date, the strains analyzed here have demonstrated the ability to be controlled by different types of antibiotics, in addition to lacking virulence genes, the data of which will be presented later.

The strains analyzed, when exposed to precursor amino acids (tyrosine, ornithine, lysine, and histidine), did not show the ability to produce biogenic amines.

### Evaluation of genes present in the DNAs of selected strains

#### Genes related to beneficial properties

With the aim of identifying genes with beneficial properties in the DNA strains studied, the beneficial genes for mucosal adhesion (*map*, *mub* and *eftu*), aggregation substance gene (*prg*), adhesion proteins (*EF*2662, *EF*1249, *EF*2380) and folate synthesis (*pab*B, *pab*C, *fol*KQ and *fol*PE), none of the 5 strains carried the respective genes in their DNA, limiting their biotechnological applications.

### Genes related to bacteriocins

The DNA of bacteria with bacteriocinogenic activity was investigated for genes responsible for bacteriocin production, regardless of species repetition. Among the bacteria, all were found to carry the *ped*PA-1 gene responsible for the production of the bacteriocin pediocin, even among the diversity of bacterial species. (Table 2). Pediocin corresponds to a class II bacteriocin, which acts on the target cell membrane by forming pores, causing cell lysis and consequent leakage of intracellular contents, leading to the death of the affected cell. Furthermore, pediocin is commonly produced by bacteria of the genus *Pediococcus*, mainly by the species *P. acidilactici* and *P. pentosaceus* (Christmann et al. 2023). However, the presence of the *ped*PA-1 gene related to pediocin production in bacteria of other species can be explained by the ability of bacterial species to transfer genetic elements horizontally between different lineages (Severn and Horswill 2023), which can also be justified by the predominance of the *P. pentosaceus* species in the Bulgarian feta cheese samples studied. Therefore, these data reinforce the bacteriocinogenic capacity of the studied species against *L. monocytogenes* strains. Similar data were obtained by Lima et al. (2024), who identified *ped*PA-1, *ent*A, and *ent*P genes related to bacteriocin production in the DNA of *E. faecium* and *Lc. garvieae* species isolated from Brazilian artisanal cheese production environments. Another study observed the presence of a gene related to pediocin production in other species different from *P. pentosaceus*. de Castilho et al. (2019a) was able to verify the presence of these genes in the DNA of *Weissella viridescens* isolated from calabrese sausage. These data reinforce the knowledge about the ability of genetic elements to be transferred horizontally between different bacterial lineages.

**Table 2:** Investigation of genes related to bacteriocin production. + positive;−negative

Strain	Genes related to bacteriocin production											
	*ent*A	*ent*B	*entL50*B	*entP*	*pedPA-1*	*plc*A	*lgnA*	*lgal*	*gak*	*gak(R1)*	*lcn972*	*lcn-gq*
*Pediococcus pentosaceus* ST0401KOC	−	−	−	−	** + **	−	−	−	−	−	−	−
*Latilactobacillus curvatus* ST0403KOC	−	−	−	−	** + **	−	−	−	−	−	−	−
*Pediococcus acidilactici* ST0412KOC	−	−	−	−	** + **	−	−	−	−	−	−	−
*Lactiplantibacillus plantarum* ST0414KOC	−	−	−	−	** + **	−	−	−	−	−	−	−
*Lacticaseibacillus paracasei* ST0110KOC	−	−	−	−	** + **	−	−	−	−	−	−	−

### Genes related to virulence

Virulence factors are undoubtedly essential agents of investigation in bacteria, especially when they come from food, since numerous genes can confer new undesirable abilities to them, increasing their resistance and ability to establish themselves in different environmental conditions (Lebreton et al. 2009). According to Table 3, the strains studied showed promising results regarding their safety.

**Table 3:** Investigation of virulence-related genes in the DNA of bacteriocinogenic bacteria. + positive; − negative

Starin	Genes related to virulence																
	*van*A	*van*B	*van*C	*van*D	*van*E	*van*G	*IS*16	*ace*	*efa*	*esp*	*asa*	*hyl*	*hdc*	*tdc*	*odc*	*cyl*A	*gel*
*Pediococcus pentosaceus* ST0401KOC	−	−	−	−	−	−	−	−	−	−	−	−	−	−	−	−	−
*Latilactobacillus curvatus* ST0403KOC	−	−	−	−	−	−	−	−	−	−	−	−	−	−	−	−	−
*Pediococcus acidilactici* ST0412KOC	−	−	−	−	−	−	−	−	-	−	−	−	−	−	−	−	−
*Lactiplantibacillus plantarum* ST0414KOC	−	−	−	−	−	−	−	+	−	−	−	−	−	−	−	−	−
*Lacticaseibacillus paracasei* ST0110KOC	−	−	−	−	−	−	+	−	−	−	−	−	−	−	−	−	−

Insertion sequences or elements (IS) contribute to greater bacterial virulence, due to providing genetic mutations (Siguier et al., 2015). IS corresponds to a small segment of DNA, carrying the transposase gene, an enzyme responsible for transposition, that is, its movement in the bacterial genome. Due to their mobility within bacterial DNA, isoisothiazolinones (IS) can interact at distinct sites, promoting genetic mutations, disruptions, and genomic rearrangements (Wan et al. 2017). IS are generally characterized negatively because they promote greater virulence in bacteria carrying and expressing this gene, providing resistance to various chemical and pharmacological agents (Firth and Skurray 2006). However, with minimal frequency, IS can be interpreted positively from an ecological perspective, considering the mutable nature of the bacteria carrying the gene, as IS can contribute to genetic variation (Kaleta et al., 2020).

The *IS*16 gene, also known as *IS*256, is widely investigated in LAB DNA, being present mainly in species related to nosocomial infections such as *E. faecalis*, *E. faecium,* and *Staph. epidermidis*. *IS*16 is used as a virulence marker for the identification and differentiation of hospital strains, since this IS is related to antibiotic resistance and the formation of bacterial biofilms (Kozitskaya et al. 2004; Werner et al. 2011). Although *IS*16 has a high prevalence in species belong to the genus *Enterococcus* (Leavis et al. 2007; Werner et al. 2011), it is possible to identify other bacterial species carrying this gene, as it is recognized that there is a sharing of genetic fragments between different bacterial lineages (Severn and Horswill 2023).

Therefore, it is plausible that *Lbs. paracasei* ST0110KOC carries the *IS*16 gene. However, although the strain in question carries the *IS*16 gene, its expression needs to be further investigated, as it may be silenced and expressed under specific conditions. *Lbs. paracasei* ST0110KOC did not show resistance to gentamicin in antibiogram tests and considering that the *IS*16 insertion element is frequently associated with the spread of resistance to aminoglycosides, such as gentamicin, the results obtained preliminarily suggest the possible absence or functional inactivity of this genetic element in the strains analyzed.

The conditions under which a bacterium establishes itself in an environment are a crucial point of investigation, so this ability is closely related to its biological performance (de Castilho et al. 2019b). Adhesion capacity occurs through the expression of proteins on the surface of the bacterial cell membrane, this being an important virulence factor, essentially for opportunistic/pathogenic bacteria, so that the anchoring of these bacteria to target cells allows invasion processes into the host system and the development of diseases (Castilho, 2016; Chajęcka-Wierzchowska et al. 2017). The *ace* gene encodes an adhesin responsible for binding to collagen types I and IV and belongs to the family of microbial surface component-recognizing adhesive matrix molecules (MSCRAMMs) (Nallapareddy et al. 2000). According to Lebreton et al. (2009), the *ace* gene was identified in several strains of *E. faecalis*, with its expression induced at a temperature of 46 °C.

The *Lpb. plantarum* ST0414KOC strain was the only one, among those analyzed, to present the *ace* gene. This gene, when expressed as previously explained, can encode a protein capable of adhering to the intestinal wall, thus favoring the colonization of the intestinal environment. However, the *Lpb. plantarum* ST0414KOC strain harbors this gene, its expression is not guaranteed, and further investigations are needed to confirm the manifestation of this potential activity.

The other genes *van*A, *van*B, *van*C, *van*D, *van*E and *van*G, *efa*, *esp*, *asa*, *hyl*, *tdc*, *odc*, *cyl*A and *gel* were not present in the other strains studied, demonstrating a positive safety profile according to genotypic tests and the absence of genes related to virulence factors.

### Mucin degradation

Investigating the mucinolytic capacity of bacteria is of paramount importance, since this ability is associated with the virulence of various pathogens. Mucin is a glycoprotein that is a constituent of mucus present on the surface of the intestinal wall of animals and humans, providing viscosity and protection (Alves 2013). Its main function is to protect intestinal cells from injuries originating from the intestinal lumen, such as shear force, digestive juices, and invasion by microorganisms (Zhou et al. 2001). In addition, intestinal mucus provides shelter for the intestinal microbiota and can also be used as an energy substrate (Derrien et al. 2010). Thus, any damage caused by its presence can lead to a deficiency in this natural defense barrier and result in possible damage.

All the strains investigated did not show mucinolytic activity when exposed to a medium without glucose. In contrast, when present in a medium containing glucose, they began to show halos around their growth, which was interpreted as positive for such activity. These results suggest that genes related to degradation capacity are activated when these bacteria are exposed to a medium containing glucose.

Mucinolytic bacteria are commonly classified as pathogenic because this ability is recognized as a virulence factor. Furthermore, bacteria that carry additional virulent factors associated with mucinolytic activity represent a greater risk to the host, since in addition to being able to express their virulent factors, they contribute to greater exposure of the intestinal wall to the conditions present in the lumen, as well as facilitating the translocation of opportunistic microorganisms (Alves 2013).

However, mucin degradation occurs naturally in the human intestinal microbiota, in which mucinolytic bacteria in the intestine undergo maturation from birth until 2 years of age (Midtvedt et al. 1994). In a way, this virulent factor does not, in itself, constitute a criterion for the microorganism's ineffectiveness, since some strains have a probiotic effect and benefit ecologically from the ability to degrade mucin (Souza 2022). As an example, *Akkermansia muciniphila* is widely used as a probiotic even though it is recognized as mucinolytic. This bacterium uses mucin as a carbon source for its nutrition and, paradoxically, has the ability to stimulate the thickening of intestinal mucus due to producing short-chain fatty acid (SCFA) metabolites, which in turn are used as an energy substrate by goblet epithelial cells responsible for mucin production (Everard et al. 2013).

Thus, although the strains analyzed showed mucinolytic activity, they may still be promising candidates for industrial applications in the food and pharmaceutical sectors, requiring further studies to investigate the influence of the mucin degradation factor.

### Characterization of the bacteriocin produced by bacteriogenic strains

#### Stability of bacteriocins present in treated supernatants under different exposure conditions

In this specific stage, the aim was also to analyze the stability of the bacteriocins produced by bacteriocinogenic strains. Thus, the supernatant of these strains was subjected to different conditions and analyzed for its activity.

The bacteriocins present in the supernatant of the bacteriocinogenic strains were stable under different temperature conditions (8 ºC–121 ºC), in the presence of chemicals, namely: sodium dodecyl sulfate, Tween 20, Tween 80, sodium chloride and skimmed milk powder, as well as at different pH levels (2.0–10.0). All supernatants containing bacteriocins from the studied strains, when subjected to the presence of Proteinase K for 1h, lost their antimicrobial activity, indicating that the substance responsible for the inhibition is of a protein nature.

The strains of *P. pentosaceus* ST0401KOC, *Ltb. curvatus* ST0403KOC, *P. acidilactici* ST0412KOC and *Lbs. paracasei* ST0110KOC did not lose their antimicrobial activity against the different serovars of *L. monocytogenes* 211, 422 and 603, in any of the variations of conditions to which they were subjected, demonstrating that they have great potential as biopreservatives.

Although bacteriocins are composed of the joining of several amino acids, these polypeptides have an extremely small size of approximately 10 kDa (Valledor et al. 2022). Due to their small structure, bacteriocins exhibit high stability when exposed to different environmental conditions. The stability of bacteriocins is widely discussed in literature, being stable when exposed to different pH values and extreme temperatures, as well as various physicochemical conditions (Carneiro et al. 2024). The results obtained here are similar to those of other authors.

According to Zhang et al. (2022), the bacteriocin produced by *B. coagulans* CGMCC 9951 proved to be stable when subjected to extreme temperature (60 ºC–121 ºC), pH (4.0–10.0) and surfactant conditions, maintaining its inhibitory activity against *L. monocytogenes*. Rwubuzizi et al. (2023) observed high stability of the bacteriocin produced by the bacteria *Lpb. plantarum*, *Ltb. curvatus*, *Lbs. paracasei*, *P. pentosaceus*, *Leu. mesenteroides* and *E. faecium*, when subjected to a wide pH range (2.0–12.0) and temperature (4 ºC–121 ºC). de Castilho et al. (2019a) points to the stability of the bacteriocin produced by *Ltb. curvatus* when subjected to temperatures from 7 ºC to 80 ºC. Data obtained by Cavicchioli et al. (2017) positively corroborates those found in this study. Analyzing the stability of the bacteriocin produced by a strain of *P. pentosaceus*, it was possible to verify a continuity of the antimicrobial effect after exposure to a wide temperature range of 4 °C–100 °C, pH 2.0–10.0, and the presence of detergent and chemicals (EDTA, SDS, NaCl, skim milk, Tween 80) against *L. monocytogenes* 211 and 422 used in this study. Zanette (2010) showed the stability of the supernatant containing bacteriocin from 4 strains of *Lpb. plantarum* against *L. monocytogenes* ATCC 7644 after heat treatment at 80 °C and 100 °C. Barbosa (2014) points to the persistence of inhibitory activity of the bacteriocin Sakacin A produced by *Latilactobacillus sakei* against *L. monocytogenes* strains after heat treatment and pH variations (4–121 °C and 2.0–10.0, respectively).

However, the bacteriocinogenic activity of the bacteria *Lpb. plantarum* ST0414KOCwas affected in all stability tests. Therefore, the bacteriocin produced by this strain are easily affected by environmental factors to which they are exposed. In contrast, the antimicrobial activity of *Lpb. plantarum* ST0414KOC was expressed when analyzed in cell growth and death curves against *L. monocytogenes* 211, 422, and 603. Therefore, these results need further study and understanding.

### Growth kinetics, acidification, and bacteriocinogenic activity of LAB

**Fig. 2:**
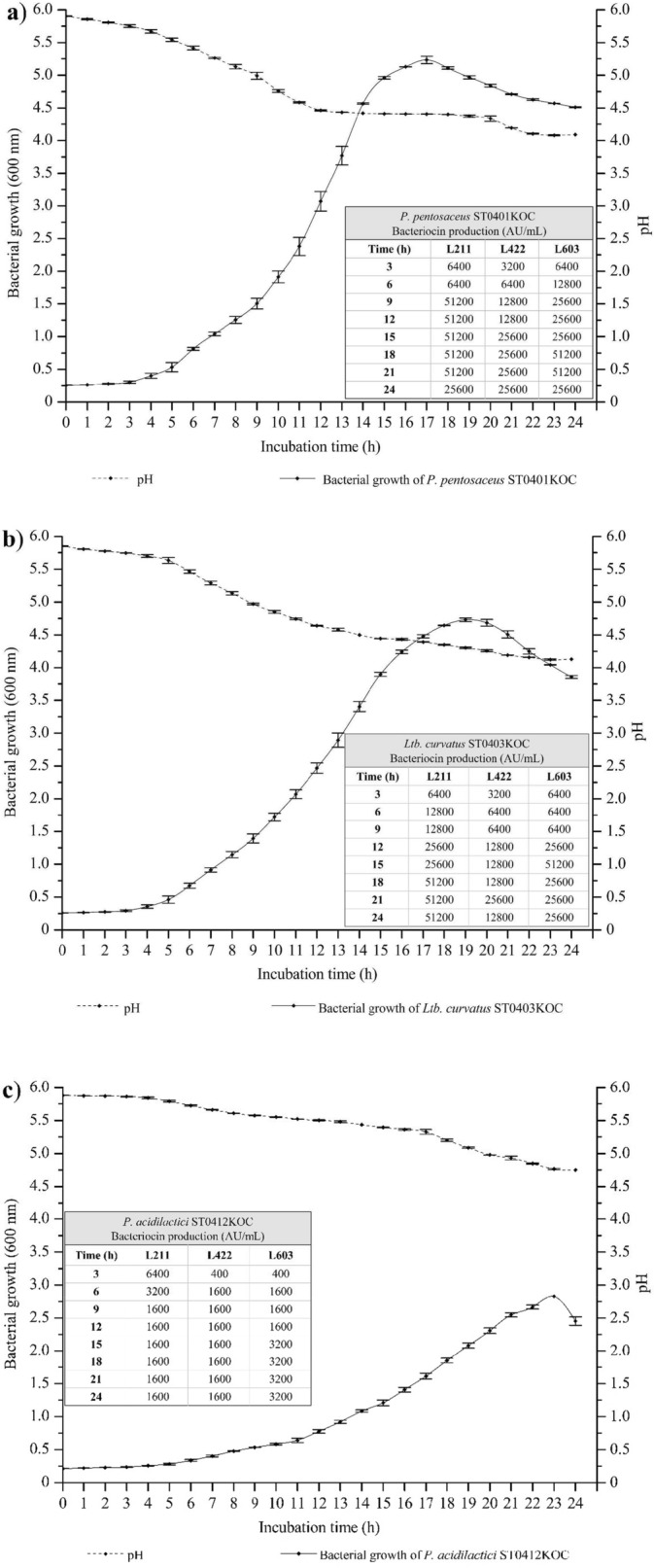

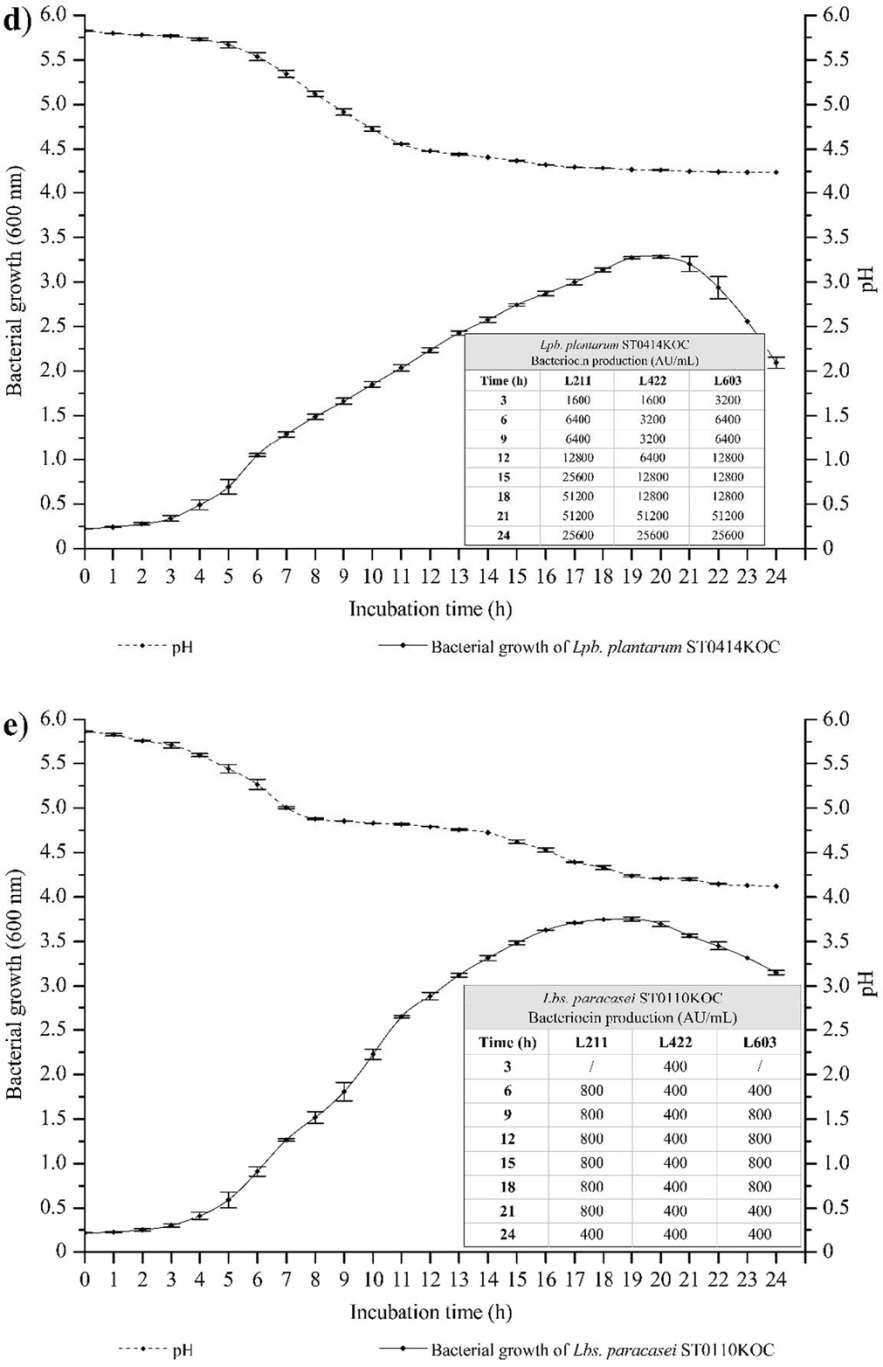
Kinetics of growth, acidification and bacteriocin production (AU/mL) by bacteriocinogenic strains. **a** *P. pentosaceus* ST0401KOC; **b** *Ltb. curvatus* ST0403KOC; **c** *P. acidilactici* ST0412KOC; **d** *Lpb. plantarum* ST0414KOC; **e** *Lbs. paracasei* ST0110KOC;

The bacteriocinogenic strains were submitted for growth kinetics studies, observing changes in cell density, pH, and bacteriocinogenic activity using three *L. monocytogenes* (211, 422, and 603) previously mentioned. Each strain exhibited individual growth behaviors and distinct bacteriocinogenic activity against different types of *L. monocytogenes*, as shown in Fig. 2.

The *P. pentosaceus* ST0401KOC strain showed 51,200 AU/mL against *L. monocytogenes* 211, with an OD of 1.504 and pH 4.99, after 9h of growth, being the strain with the highest activity in the shortest growth time. *P*. *acidilactici* ST0412KOC showed maximum bacteriocin values of 6400 AU/mL after 3h of growth against *L. monocytogenes* 211 (OD 0.236, pH 5.86). *Ltb. curvatus* ST0403KOC showed maximum bacteriocinogenic activity levels of 51,200 AU/mL after 15h of growth against *L. monocytogenes* 603 (OD 3.898, pH 4.44). Barbosa et al. (2014) showed the production of 1600 AU/mL by *Ltb. sakei* MBSa1 when incubated at 25 °C. Furthermore, its bacteriocin proved effective in inhibiting the three different serovars of *L. monocytogenes* studied here. Cavicchioli et al. (2017) reported the production of 3200 AU/mL by *P. pentosaceus* ST65ACC isolated from Brazilian artisanal cheese against *L. monocytogenes* 422. Zanete (2010) recorded 800 AU/mL of bacteriocin produced by different strains of *Lct. plantarum*. These results reinforce the ability of LAB to synthesize significant quantities of bacteriocins, consistent with the performance of the bacteriocinogenic strains used in this study, highlighting the high values of bacteriocins produced expressed in AU/mL.

Other strains showed good results, but with a greater presence of bacteriocins when there is a longer incubation period. Some bacterial strains require a longer period of time to reach their maximum production limit and antimicrobial activity. According to data obtained in this study,

The strains *Lbs. paracasei* ST0110KOC was the strain that showed the least bacteriocinogenic activity, with maximum value of 800 AU/mL, against *L. monocytogenes* 211 (OD 0.911, pH 5.26). This strain did not demonstrate high antimicrobial capacity against *L. monocytogenes* but rather slowed its growth.

The strains isolated from Bulgarian cheese samples demonstrated bactericidal activity, since after inoculation (hour 3) of the treated supernatant containing bacteriocin in the broth containing *L. monocytogenes*, it prevented its growth throughout the subsequent experimental period (12h incubation). This can be observed in the cell death curves of *L. monocytogenes* presented below, where the pathogen did not demonstrate any degree of ascendancy after the presence of supernatant from strains originating from Bulgarian cheese.

However, it is possible to observe that the strain *Lbs. paracasei* ST0110KOC presented low levels of bacteriocinogenic activity as already mentioned (maximum of 800 AU/mL) and the strain caused a slowdown in the growth of the pathogen when compared to the control, thus having a bacteriostatic action, according to data to be discussed sequentially. Results related to growth kinetics can be observed in Fig. 2.

### Cell death kinetics of *L. monocytogenes* by treated supernatants containing bacteriocins

The treated supernatants containing bacteriocin from the bacteriocinogenic strains were analyzed for antimicrobial capacity against the previously used *L. monocytogenes* 211, 422, and 603 strains. These were inoculated after 3h of growth of each *L. monocytogenes*, measuring their growth in an OD at 600 nm every hour for 12h.

The treated supernatants of the bacteriocinogenic strains *P. pentosaceus* ST0401KOC, *Ltb. curvatus* ST0403KOC, *P. acidilactici* ST0412KOC, *Lpb. plantarum* ST0414KOC, when added to broths containing *L. monocytogenes* (at approximative cells density of 10^5^ CFU/mL), showed an inhibitory effect during the subsequent 9h of the experiment, in which there were no ascending curves after inoculation at 3h of incubation, demonstrating a bactericidal action.

Although the strains showed antimicrobial activity against *L. monocytogenes* 211, 422, and 603, each bacteriocin produced behaved specifically against each serovar of the pathogen. Even though the bacteriocinogenic strains showed high levels of bacteriocin produced, the amounts inoculated of each supernatant containing bacteriocin were consistent with the respective final values, since the treated supernatant used was from 24h of growth. Thus, it was possible to observe different values in AU/mL expressed against each serovar of *L. monocytogenes* analyzed, as shown in Fig. 2.

**Fig. 3:**
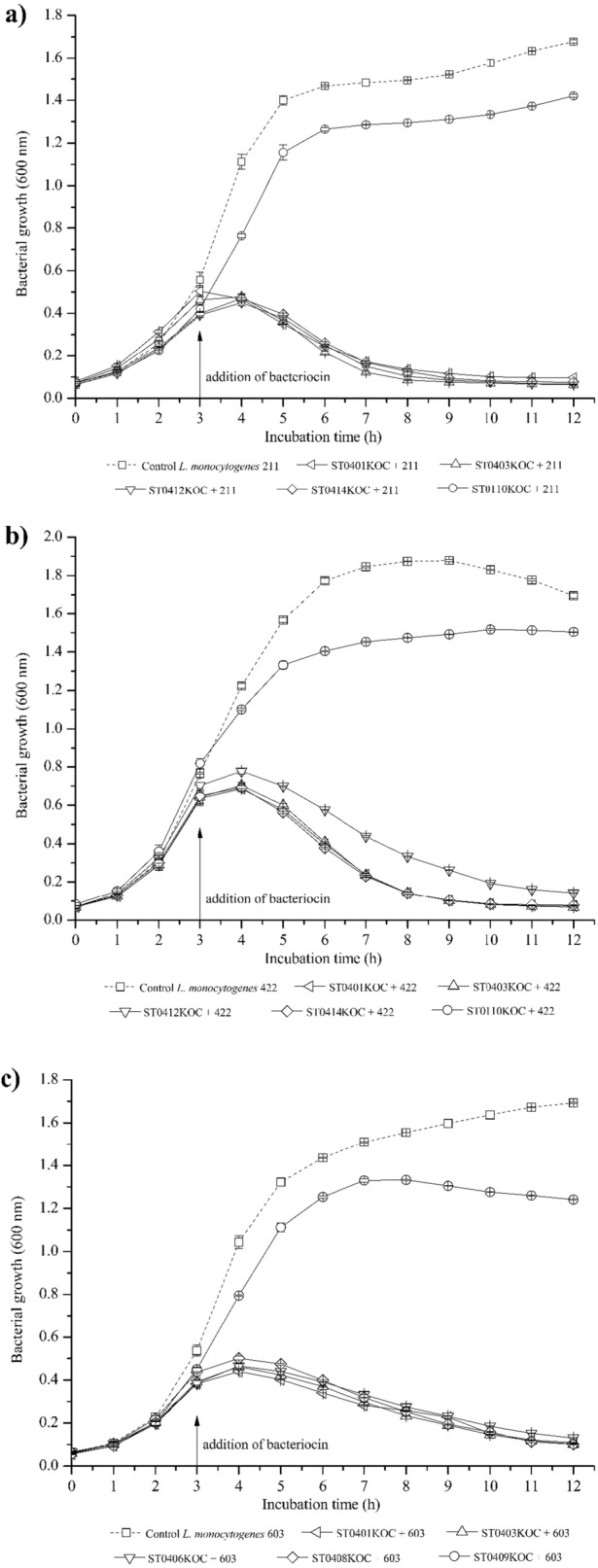
Inhibition of different serovars of *L. monocytogenes* during 12h. The arrow represents the inoculation time of the treated supernatant of each bacteriocinogenic strain (individual concentrations in AU/mL). **a**) bacteriocinogenic supernatants + *L. monocytogenes* 211 serovar 4b; **b** bacteriocinogenic supernatants + *L. monocytogenes* 422 serovar 1/2c; **c** bacteriocinogenic supernatants + *L. monocytogenes* 603 serovar 1/2b

These small cationic proteins have a general mode of action that consists of causing injury to the cell membrane of the target cell, inhibiting peptidoglycan biosynthesis or directly forming pores in the cell membrane, leading to cell death as represented in Fig. 3 (Baindara et al. 2018; Bhattacharya et al. 2022). It is worth noting that some bacteriocins demonstrate anticancer activity even without promoting the rupture of the plasma membrane, acting through intracellular mechanisms. These molecules can be targeted to the mitochondria of tumor cells, interacting with their membrane (which, due to the high concentration of anionic lipids, has a negative charge), or even directly interfere with the cell cycle, compromising proliferative processes (Punj et al. 2004).

Bacteriocins are compared to antibiotics because of their antimicrobial activity. However, they differ in their specificity of action, acting preferentially on microorganisms phylogenetically close to the producing bacterium (Chikindas et al. 2018). Although these antimicrobials act on closely related microorganisms, some LAB produce bacteriocins that may have a broader spectrum of inhibition, such as against Gram-negative bacteria, yeasts, fungi, and viruses (Todorov et al. 2019).

These antimicrobial peptides have received considerable attention from the food industry because they provide greater safety for food and consumers (Hakim et al. 2023). Foods fermented by LAB are natural sources of bacteriocins, so this metabolite is synthesized simultaneously in the fermentation process (Freire et al. 2021). Although several LAB exhibit bacteriocinogenic activity, this property does not interfere with starter cultures used in food production, restricting its action only to specific target microorganisms (Carneiro et al. 2024).

In addition to their application in food production, bacteriocins produced by LAB can also be added to food packaging in order to preserve the food and prevent possible contamination with pathogenic or spoilage microorganisms (Thapar and Salooja, 2023). Silva et al. (2023) observed that packaging containing enterocin could inhibit the presence of *L. monocytogenes*. This result demonstrates the reduction of food contamination when enterocin is incorporated into the packaging, providing greater microbiological safety for the product.

As an example, a study conducted by Cavicchioli et al. (2017) identified LAB of the genus *Enterococcus hirae* and *Pediococcus pentosaceus* with antimicrobial capacity against *Listeria innocua* and *L. monocytogenes*. Lima (2024) found bacteriocinogenic activities of *Enterococcus faecium* and *Lactococcus garvieae* isolated from different surfaces of a Brazilian cheese factory against *L. monocytogenes*.

It is also noteworthy that in addition to their antimicrobial activity, numerous bacteriocins have demonstrated potential anticancer effects. Healthy cells have a neutral cell membrane due to being rich in phosphatidylcholine and sphingomyelin. However, tumor cells begin to express anionic molecules in their plasma membrane, resulting from alterations in their lipid and protein composition. As a result of these alterations, the cell membrane of these cells becomes composed of phosphatidylserine, O-glycosylated mucins, sialic gangliosides, and heparin sulfates, thus acquiring a negative charge (Wang et al. 2024). This change in charge favors interaction with bacteriocins, which, being cationic, bind to these cells selectively, triggering the formation of pores in the cell membrane and consequent leakage of its contents, or even inducing cell death in cytoplasmic interactions (Aghamiri et al. 2021; Riedl et al. 2011; Wang et al. 2024).

The analyzed strains, as previously stated, demonstrated high antimicrobial activity, with maximum bacteriocinogenic expressions, in most cases, of 51,200 AU/mL. These results reinforce the efficient bacteriocinogenic ability of the analyzed strains.

Similar results were obtained by Lima et al. (2024). *E. faecium* and *Lc. garvieae* strains isolated from cheese manufacturing environments showed bacteriocinogenic properties against *L. monocytogenes* ATCC 7644. After adding the treated supernatant containing bacteriocin after 3h of pathogen growth (logarithmic phase), its growth was inhibited for the following 9h, demonstrating a wide difference in growth when compared to the control.

Ceruso et al. (2021) reinforce the antimicrobial properties of bacteriocins against *L. monocytogenes*. In the study, a pediocin-producing strain of *Lpb. plantarum* was able to significantly inhibit or delay the growth of *L. monocytogenes* for a period of 24h. Furthermore, the authors observed that the application of thermophilin 110, at concentrations greater than 1280 AU/mL, resulted in a sustained bactericidal effect throughout the entire experimental interval. Cavicchioli et al. (2017) demonstrate that the bacteriocin produced by a *P. pentosaceus* ST65ACC strain, when added in the logarithmic phase after 3h of pathogen growth, is able to inhibit the growth of two different serovars of *L. monocytogenes* (211, serovar 4b; and 422, serovar 1/2c) over 9h of incubation, corroborating the bactericidal effect observed for the *P. pentosaceus* ST0401KOC strain used in this study.

On the other hand, the supernatant of the strain *Lbs. paracasei* ST0110KOC showed a slowing of bacterial growth. Even so, the optical density values when compared with the control are close and, depending on the growth time, relatively equal, a condition interpreted as a bacteriostatic effect. This can be explained by the low antimicrobial activity expressed in previous analyses (Fig. 2), with its maximum activity being 800 AU/mL. The results regarding the kinetics of cell death by each bacteriocinogenic microorganism are shown further on in Fig. 3**.**

Considering the proposed objectives and the results achieved in this study, it is concluded that there is a predominance of LAB with beneficial properties in the Bulgarian feta cheese and Brazilian *prato* cheese samples analyzed. Among the 146 isolates obtained from the cheese samples, following the selection process according to the set of safety characteristics, properties of interest and identification by 16S, it was possible to obtain 5 bacteriocin-producing strains. The 5 strains identified as *P. pentosaceus* ST0401KOC, *Lct. curvatus* ST0403KOC, *P. acidilactici* ST0412KOC, *Lpb. plantarum* ST0414KOC and *Lbs. paracasei* ST0110KOC, in addition to being considered safe in phenotypic tests, also proved to be safe when subjected to additional genotypic tests according to the tests performed so far. In the set of 24 genes investigated related to antibiotic resistance, virulence, and toxin production, only the 3 undesirable genes *ace* and *IS*16 were present in the DNA of *Lpb. plantarum* ST0414KOC and *Lbs. paracasei* ST0110KOC, respectively. Although these genes are present, they do not show their expression.

When the bacteriocinogenic capacity of the studied strains was analyzed, a high prevalence of the *ped*PA-1 gene, associated with pediocin production, was observed, present in 93% of the strains with antimicrobial activity. The strains with this property showed a broad spectrum of inhibition against different serovars of *L. monocytogenes*, in addition to inhibiting its growth in its logarithmic phase during 9h of incubation.

This work provides contribution to understanding and scientific development focused on alternatives in the control of the pathogen *L. monocytogenes* using bacteriocins. Therefore, the strains *P. pentosaceus* ST0401KOC, *Lct. curvatus* ST0403KOC, *P. acidilactici* ST0412KOC, *Lpb. plantarum* ST0414KOC, and *Lbs. paracasei* ST0110KOC obtained in this study, due to their anti-*Listeria* properties, promote promising alternatives as biocontrol agents. Added to the demonstrated safety, these strains stand out as potential candidates to be probiotic, as well as for future use by the food and pharmaceutical industries.

## Supplementary Information

Below is the link to the electronic supplementary material.

Supplementary file1 (DOCX 18 kb)

## Data Availability

No datasets were generated or analysed during the current study.
