# Supplementary material for: Bacteriocinogenic properties of lactic acid bacteria isolated from Bulgarian feta cheese and Brazilian prato cheese against Listeria monocytogenes: Bacteriocin, an ally in the control of listeriosis
Source: Antonie Van Leeuwenhoek. 2026 May 12;119(6):124. doi: 10.1007/s10482-026-02335-5 (PMC13167866; doi:10.1007/s10482-026-02335-5)
Supplement: Supplementary file 1 — Supplementary file1 (DOCX 18 kb) [file 10482_2026_2335_MOESM1_ESM.docx]

**Supplementary**

Supplementary Table 1: Primers used to investigate the presence of target genes in the DNA of the studied strains, analyzed according to their respective size.

| Targeted gene | Size of expected amplicon, bp | Reference |
| --- | --- | --- |
| ***Genes related to differentiation and identification*** | | |
| **(GTG)_5_** |  | de Castilho *et al.* (2019a) |
| ***BSF 8/20*** | 1518 | Héquet *et al*. (2007) |
| ***Genes related to bacteriocin production*** | | |
| ***entA*** | 452 | Fugaban *et al.* (2021b) |
| ***entB*** | 159 |  |
| ***entL50B*** | 216 |  |
| ***entP*** | 216 |  |
| ***pedPA-1*** | 1238 | Todorov *et al*. (2010) |
| ***plc A*** | 245 | Todorov *et al.* (2017) |
| ***lgnA*** | 205 | Maldonado Barragán *et al.* (2013) |
| ***lgal*** | 261 |  |
| ***gak*** | 341 | Telke *et al.* (2019) |
| ***gak(R1)*** | 350 |  |
| ***lcn972*** | 232 | Mirkovic *et al.* (2015) |
| ***lcn-gq*** | 382 |  |
| ***Beneficial genes*** | | |
| ***pab*B** | 1666 | Kim *et al.* (2022) |
| ***pab*C** | 1850 |  |
| ***fol*-KQ** | 790 |  |
| ***fol*-PE** | 1350 |  |
| ***map*** | 200 | de Castilho *et al.* (2019b) |
| ***mub*** | 200 |  |
| ***eftu*** | 200 |  |
| ***prg*** | 3917 |  |
| ***ef1249*** | 1712 |  |
| ***ef2662*** | 1121 |  |
| ***ef2380*** | 1268 |  |
| ***Genes related to vancomycin resistance*** | | |
| ***van*A** | 231 | Fugaban *et al.* (2021a) |
| ***van*B** | 330 |  |
| ***van*C** | 402 |  |
| ***van*D** | 500 |  |
| ***van*E** | 513 |  |
| ***van*G** | 519 |  |
| ***Genes related to virulence properties*** | | |
| ***IS*16** | 547 | Werner *et al.* (2011) |
| ***ace*** | 1008 | Martín Platero *et al.* (2009) |
| ***efa*** | 688 |  |
| ***esp*** | 510 | Vankerckhoven *et al.* (2004) |
| ***asa*** | 375 |  |
| ***hyl*** | 276 |  |
| ***hdc*** | 367 | de Las Rivas *et al.* (2005) |
| ***tdc*** | 924 |  |
| ***odc*** | 1446 |  |
| ***cylA*** | 688 | Vankerckhoven *et al.* (2004) |
| ***gel*** | 213 |  |

Supplementary Table 2: Distinction of isolates after identification by 16s and *rep*PCR. QP1- Cheese Plate; QB2- Bulgarian feta-type cheese; QB3- Bulgarian feta-type cheese; QB4- Bulgarian feta-type cheese.

| Isolates | Sample | Species | Nº Gel | Group |
| --- | --- | --- | --- | --- |
| ST0110KOC | QP1 | *Lacticaseibacillus paracasei* | **12** | **E** |
| ST0158KOC | QP1 | *Lacticaseibacillus paracasei* | **13** | **E** |
| ST0401KOC | QB4 | *Pediococcus pentosaceus* | **1** | **A** |
| ST0402KOC | QB4 | *Pediococcus pentosaceus* | **2** | **A** |
| ST0403KOC | QB4 | *Latilactobacillus curvatus* | **3** | **B** |
| ST0406KOC | QB4 | *Pediococcus pentosaceus* | **11** | **A** |
| ST0407KOC | QB4 | *Pediococcus pentosaceus* | **4** | **A** |
| ST0408KOC | QB4 | *Pediococcus pentosaceus* | **9** | **A** |
| ST0410KOC | QB4 | *Pediococcus pentosaceus* | **5** | **A** |
| ST0412KOC | QB4 | *Pediococcus acidilactici* | **6** | **C** |
| ST0414KOC | QB4 | *Lactiplantibacillus plantarum* | **7** | **D** |
| ST0415KOC | QB4 | *Pediococcus pentosaceus* | **10** | **A** |
| ST0420KOC | QB4 | *Pediococcus pentosaceus* | **8** | **A** |
